# Peritoneal melanosis: case report and literature review

**DOI:** 10.1093/jscr/rjag501

**Published:** 2026-06-27

**Authors:** Yasmine Barbirou, Haroun Guermazi, Malek Ben Younes, Salma Bradai, Adnen Chouchen, Sarra Ben Rejeb

**Affiliations:** Pathology Department, Internal Security Forces Hospital, La Marsa, 2078, Tunisia; General Surgery Department, Internal Security Forces Hospital, La Marsa, 2078, Tunisia; General Surgery Department, Internal Security Forces Hospital, La Marsa, 2078, Tunisia; General Surgery Department, Internal Security Forces Hospital, La Marsa, 2078, Tunisia; General Surgery Department, Internal Security Forces Hospital, La Marsa, 2078, Tunisia; Pathology Department, Internal Security Forces Hospital, La Marsa, 2078, Tunisia

**Keywords:** melanosis, peritoneum, pigmentation disorders, ovarian cysts, hernia, umbilical, incidental findings

## Abstract

Peritoneal melanosis is a rare benign pigmentation disorder of the peritoneum, often discovered incidentally during surgery, alarmingly mimicking metastatic melanoma. We present the case of a 35-year-old female, who underwent emergency repair for a strangulated umbilical hernia. Intraoperatively, a 0.5 cm pigmented nodule was incidentally found on the pelvic peritoneum, alongside a similar ovarian lesion. An excisional biopsy was performed to rule out malignancy. Histopathological examination revealed fibro-adipose tissue with a dense inflammatory infiltrate and pigment-laden macrophages, confirming peritoneal melanosis. With fewer than 20 cases reported in the literature, this condition poses a significant intraoperative diagnostic challenge. Awareness is crucial for surgeons and pathologists to prevent the misdiagnosis of melanoma and prompt appropriate investigation for frequently associated occult ovarian or gastrointestinal lesions.

## Introduction

Peritoneal melanosis is a rare benign condition, characterized by focal or diffuse pigmentation of the peritoneum, often appearing as staining, speckling, or tumour-like nodules [1, 2]. It is commonly incidentally discovered during laparotomy [3]. Most cases are associated with other conditions, especially congenital cysts [1, 4]. Though, it must be distinguished from metastatic melanoma or colonic malignancy [4].

To the best of the author’s knowledge, fewer than 20 cases have been reported in the literature [3].

We herein described another rare case of pelvic peritoneal melanosis incidentally discovered during umbilical hernia repair.

## Case report

A 35-year-old female patient, with a gynecological history of two cesarean sections, presented to the emergency department with an acute abdominal pain. Clinical examination revealed an umbilical hernia, with early signs of strangulation. Umbilical hernia repair was performed.

**Figure 1:**
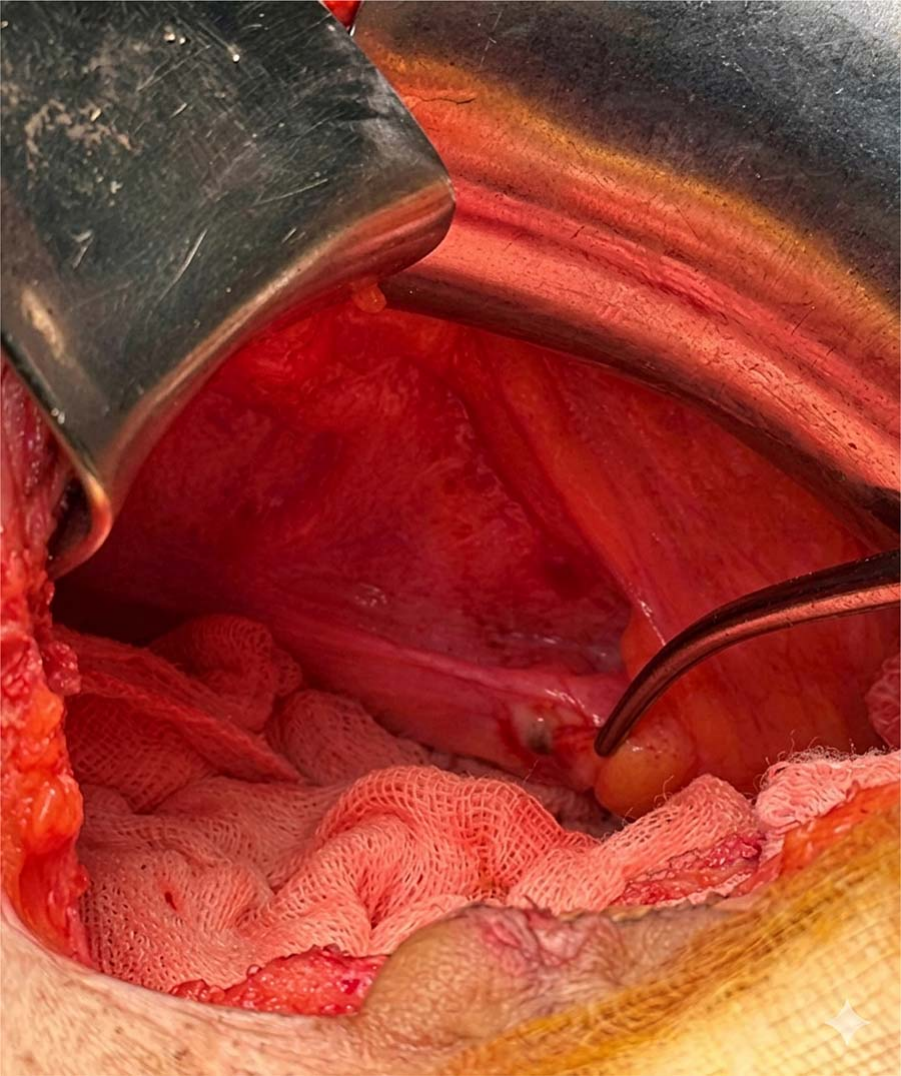
Per-operative findings on the pelvis: An incidental pigmented peritoneal nodule.

**Figure 2:**
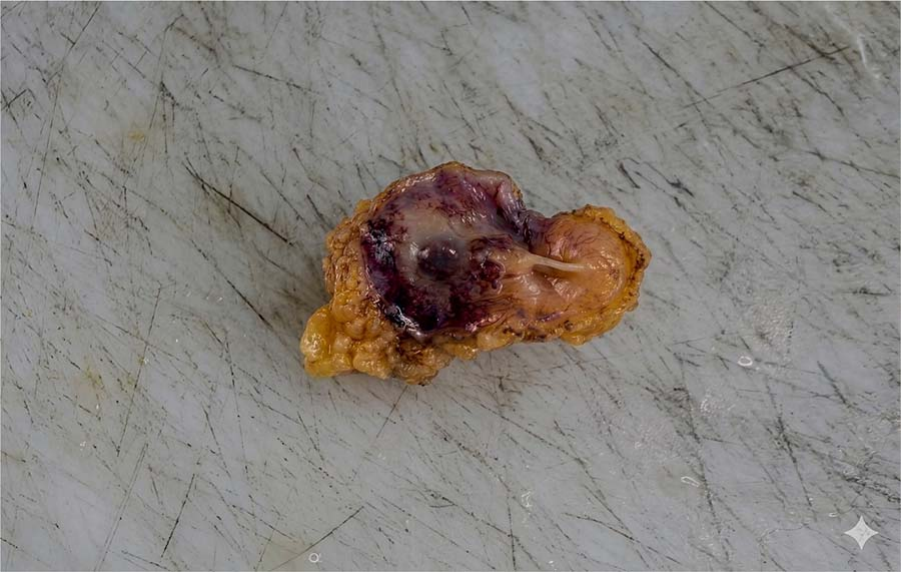
Macroscopic appearance of the excised peritoneal nodule: A whitish background, centered by brown pigmentation and circumscribed by hemorrhagic stippling.

During surgical exploration, a 0.5 cm pigmented nodule was identified on the pelvic peritoneum (Fig. 1), along with a similar pigmented lesion on the right ovary. The dark pigmentation raised strong suspicion for metastatic melanoma or other malignant peritoneal deposits, particularly in the context of an emergency setting where full staging was not available. Given the uncertainty and the need to rule out malignancy, an excisional biopsy of the peritoneal lesion was firstly performed. Macroscopically, the specimen measured 2.5 × 1.5 × 1 cm and consisted of fibro-fatty tissue, centered by an indurated and poorly circumscribed brownish pigmented nodule (Fig. 2).

**Figure 3:**
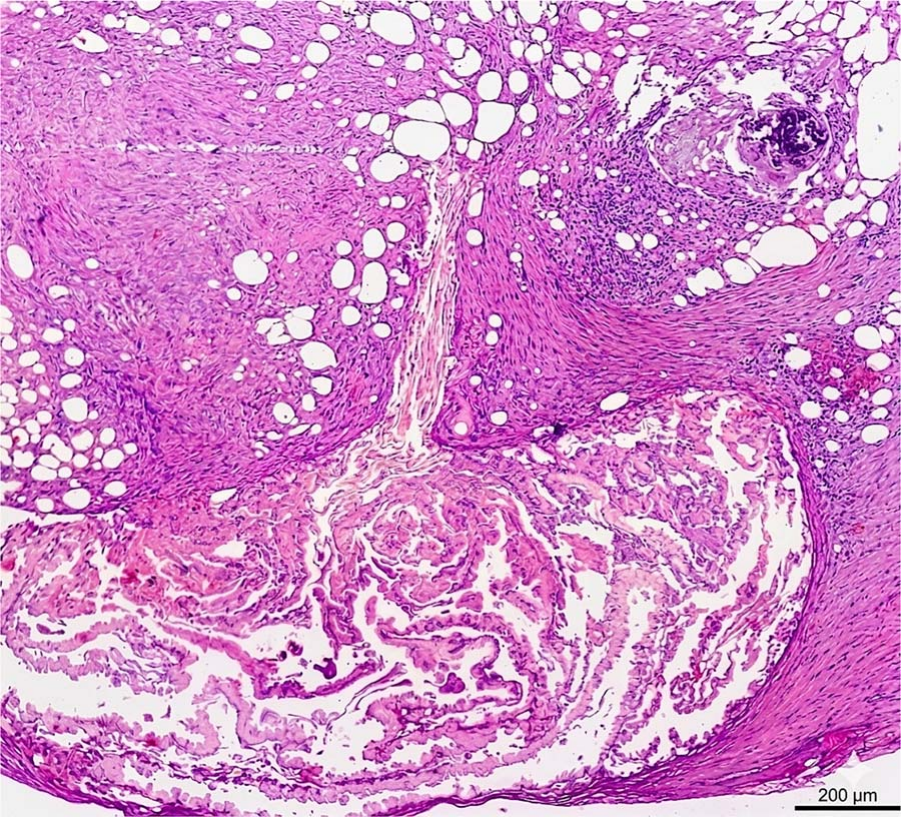
Microscopic appearance of the peritoneal pigmented lesion at low magnification (×40) with standard hematoxylin and eosin staining: Deposits of pale, eosinophilic and light-yellowish material, organized in a festooned acellular pattern underneath the mesothelium, surrounded by inflammation and focal calcification.

**Figure 4:**
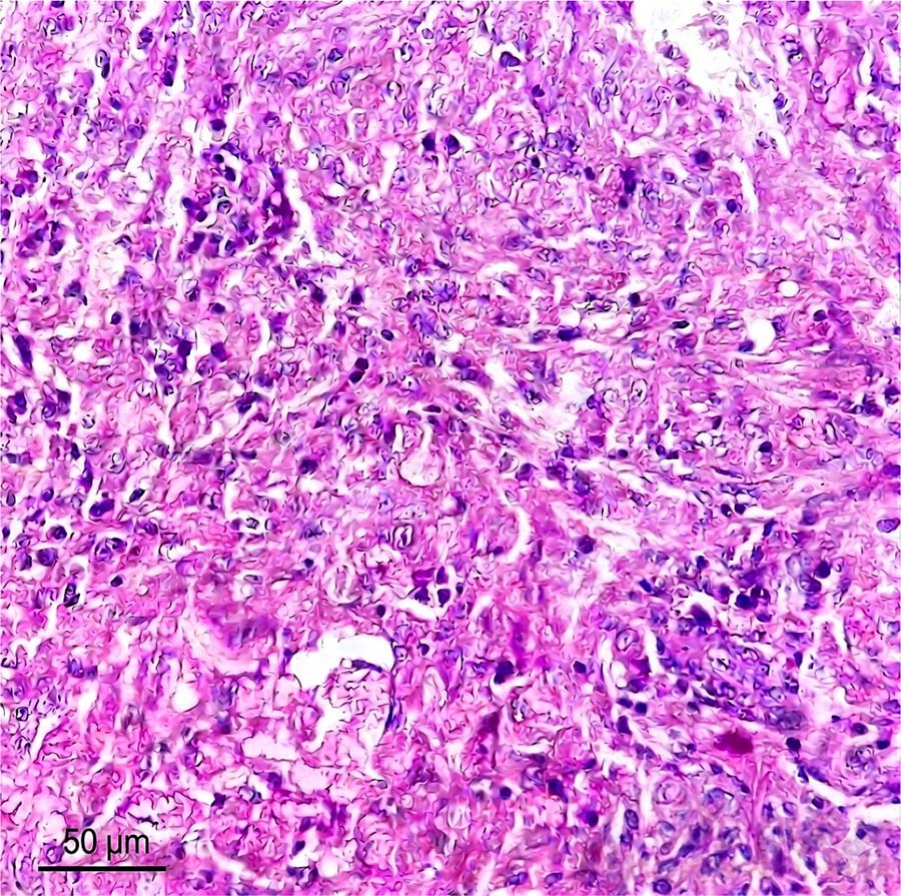
Microscopic appearance of the peritoneal pigmented lesion at high magnification (×400) with standard hematoxylin and eosin staining: Dense inflammatory infiltrate rich in macrophages, appearing either foamy or dotted with yellowish-brown pigments, surrounding the material deposits.

Microscopic examination revealed fibro-adipose tissue with marked inflammatory changes, pigment-laden macrophages containing yellowish-brown granules, and deposits of pale eosinophilic, partially calcified material in a festooned pattern beneath the mesothelial lining (Figs 3-4). There was no evidence of atypical melanocytic cells, endometriosis, deciduosis, or malignancy. These features were consistent with peritoneal melanosis.

At 3-month follow-up, the patient remained asymptomatic with no clinical or radiological evidence of malignancy on gynecological ultrasound and abdominal examination. Further imaging was not pursued given the benign histology and absence of concerning features.

## Discussion

Peritoneal melanosis is a very rare and benign peritoneal condition, with fewer than 20 well-documented cases described in the literature (Table 1) [3].

**Table 1:** Table summarizing the documented cases of peritoneal melanosis in the literature from 1948 to 2025

**Author (Year)**	**Gender**	**Age**	**Medical history**	**Clinical presentation**	**Radiological examinations**	**Peroperative findings**	**Associated diagnosis**	**Treatment**	**Follow-up/ Outcomes**
Esayas B, *et al.* (2025) [3]	Female	71 years	Gravida 12 Para 12	- Cachexia. - Significant abdominal distension with ascites.	Abdominopelvic US ^**a**^+ CT^**a**^ scan: A large abdominopelvic mass with solid and cystic lesions and metastatic deposits on the omentum.	Diffuse dark pigmentation involving the greater omentum, visceral peritoneum and serosa of the large and small bowel segments.	Mucinous adenocarcinoma of the ovary	- 1^st^ surgery: Unilateral salpingo-oophorectomy. - 2^nd^ surgery: Total abdominal hysterectomy. - Adjuvant chemotherapy: Carboplatin + paclitaxel.	- Smooth post-operative course. - Good performance status 8 months after the second surgery.
Trifina-Mikosch E, *et al.* (2023) [14]	Female						Mucinous cystadenoma of the ovary		
Mhand M, *et al.* (2022) [5]	Female	67 years	- Gravida 6 Para 5. - Combined oral contraception. - Type 2 diabetes. - Hypertension.	- Altered general health. - Abdominal distension with diffuse dullness and chronic hypogastric pain.	CT scans: A locally advanced right pelvic ovarian mass with suspected invasion of the uterus and anterior rectal wall, peritoneal carcinosis and abundant pleural effusion.	Several dark-brown/black nodules on the peritoneum, parietal wall, and pelvis.	Serous carcinoma of the ovary	- Neoadjuvant chemotherapy: 7 courses of carboplatin + paclitaxel + bevacizumab. - Surgery: Hysterectomy + appendectomy + omentectomy.	- Simple post-operative course. - Doing well with no signs of recurrence after 1 year of clinical and radiological surveillance.
Barghash M, *et al.* (2021) [1]	Male	86 years	- Hypertension. - Chronic obstructive pulmonary disease.		CT scan: A large right inguinal hernia containing small bowel loops. Lower GI^**a**^ endoscopy: A 25 mm distal sigmoid polyp.	Widespread black spots on the hernial sac during hernia repair.	Adenocarcinoma of the distal sigmoid	Endoscopic resection	Regular CEA^**a**^ levels, lower gastrointestinal endoscopy and CT scan in six months.
Sim KK, *et al.* (2021) [12]	Male	49 years	Melanoma of the right shoulder with a solitary right axillary node metastasis	Right supra-clavicular nodal relapse	PET ^**a**^ scans: Disease progression involving a new splenic lesion and a right axillary lesion.	Diffuse black speckling on the peri-splenic omental surface, the anterior abdominal wall and the diaphragmatic surface of the left upper region.	Metastatic melanoma involving the spleen	- Surgery: Right supra-clavicular node and neck dissection + Laparoscopic splenectomy + Hookwire excision of axillary lesions - Adjuvant radiotherapy. - Targeted therapy: BRAF/ MEK inhibitors. - Immunotherapy: Immune checkpoint inhibitor.	PET scan after 4 cycles of low-dose immunotherapy.
Zhao C, *et al.* (2021) [13]	Female	49 years				Diffuse brown-black pigments on pelvis, intestinal tube, omentum, mesentery and diaphragm.	Mucinous cystic tumor of the ovary	Laparoscopic salpingo-oophorectomy	
Chang E, *et al.* (2015) [11]	Female	23 years	Anemia.	Episodes of fresh blood in the stools.	Elective colonoscopy: A 5 cm sessile rectal polyp.	Diffuse black pigmentations on the parietal peritoneum, greater omentum, mesenteric lymph nodes and ovaries.	Adenocarcinoma of the rectum	Laparoscopic anterior resection of the rectum.	- Uneventful surgery. - Complete healing and no cancer recurrence during post-discharge follow-up. - No progression or remission of peritoneal melanosis.
Jamkhandi DM, *et al.* (2014) [4]	Female	20 years	-Consanguineous marriage. - Spontaneous abortion at 12 weeks of gestation. - 2^nd^ gravida.	Pre-term labor pains and fetal distress at the 35^th^ week of gestation.		Blackish-green thick viscous fluid and dark black pigmented patches in the peritoneal cavity	Dermoid cyst of the ovary	Emergency cesarean section + Right salpingo-oopherectomy.	Both mother and baby were doing well at 8 months post-partum.
Kim SS, *et al.* (2010) [6]	Female	68 years	- Gravida 7 Para 7. - Anemia.	Lower abdominal pain and distension.	Pelvic CT scan: A huge multi-cystic mass on the right ovary and a 3,7 cm well-enhancing intraluminal mass at the rectosigmoid junction.	India-ink-colored pigmentation in the peritoneum, omentum, bladder, mesentery and the surfaces of both the ovarian mass and colonic serosa.	Mucinous cystadenoma of the ovary + Adenocarcinoma of the colon	Modified radical hysterectomy with bilateral salpingo-oophorectomy + Low anterior resection of the colon and bilateral pelvic lymph node dissection.	
Liu Y li, *et al.* (2007) [7]	Female	14 years		Abdominal pain and tenderness around the navel and the right iliac fossa.	US: A 5,2 cm mass on the right adnexa, possibly an ovarian dermoid cyst.	Concentration of omentum in the right lower abdomen with scattered black spots, similar pigment spots on the appendix and multiple black, villous-like growths on the small intestine and mesentery.	Dermoid cyst of the ovary	Emergency laparotomy	Discharged 10 days post-surgery with good recovery.
Kim NR, *et al.* (2002) [8]	Female	23 years		Palpable pelvic mass	Pelvic US: A 30 cm unilocular cystic mass in the right ovary.	Dark black pigmented patches involving the entire omentum, peritoneum, ovarian surface, and appendix.	Serous cystadenoma of the ovary	Right salpingo-oophorectomy + Appendectomy	- Uneventful postoperative course. - Remained well throughout a 5-year-and-a-half follow-up.
Jaworski RC, *et al.* (2001) [2]	Female	27 years	Gravida 1 Para 1	Severe pain with mild to moderate tenderness in the left iliac fossa with guarding and rebound	US: A 9 cm complex left adnexal mass, likely a dermoid cyst.	Numerous areas of black discoloration on the omentum, pelvic peritoneum, serosa of the sigmoid colon and pouch of Douglas.	Dermoid cyst of the ovary	Left salpingo-oophorectomy.	
Nada R, *et al.* (2000) [15]	Female	1 year and a half					Enteric duplication cyst		
De la Torre Mondragón L, *et al.* (1997) [9]	Male	6 months		Asymptomatic, firm and mobile mass in the left hypochondrium.	US: 2 cystic lesions between the stomach and left kidney. GI series: Medial displacement and compression of the greater curvature of the stomach.	Diffuse black pigmentation of the peritoneum with nodular patches and loose adhesions	Gastric triplication	Laparotomy with total resection of the masses + Appendectomy	Asymptomatic 3 years after surgery.
Jung YC, *et al.* (1996) [17]	Female	2 years					Enteric duplication cyst		
Drachenberg C, *et al.* (1990) [16]							Melanotic peritoneal cyst		
Fukushima M, *et al.* (1984) [10]	Female	28 years	Gravida 3 Para 3	- Menstrual delay postpartum. - Acute abdominal and pelvic pain and fullness. - A large pelvic mass 3 months postpartum.	Intravenous pyelogram: Ureteral displacement and a large pelvic soft tissue density with areas of calcification. US: A large multilocular cystic lesion.	Tar-colored/ black pigment deposits of 1 to 5 cm covering the surfaces of the ovaries, uterus, fallopian tubes, omentum, bowel serosa, appendix and peritoneum.	Dermoid cyst of the ovary	- Conservative surgery: Left salpingo-oophorectomy + Appendectomy. - 2^nd^-look laparoscopy (after 5 months): Persistent pigmented lesions were smaller or had been completely reabsorbed.	- Uneventful postoperative recovery. - No evidence of recurrence 3 and a half years after the initial laparotomy.

^a^Abbreviations: US: Ultrasound, CT: Computed tomography, GI: Gastrointestinal, CEA: Carcinoembryonic antigen, PET: Positron emission tomography.

The mean age of patients was 35 years and ranged from 6 months to 86 years. Consistent with the findings in our reported case, a female predominance was noted (81%) (male to female ratio = 0.23).

Peritoneal melanosis is most often an incidental finding, discovered during surgery or laparoscopy for other conditions [3]. When symptoms are present, they are usually related to the underlying disease rather than the melanosis itself. The most common presenting manifestations include abdominal pain and/or distension (38%) [2–10]. In the present case, the acute abdominal pain was attributed to the strangulated umbilical hernia and not directly to the peritoneal melanosis.

Although no radiological investigations were performed in the present case, literature data indicate that imaging in similar cases were non-specific and revealed abdominal and/or pelvic masses (73%) in most cases [1–3, 5–12].

Peritoneal melanosis shows a wide anatomical distribution within the peritoneal cavity. The most affected sites are the visceral peritoneum of the small and large bowel (81%), greater omentum (50%), ovarian surface (25%), mesentery (19%), parietal abdominal peritoneum (19%), parietal pelvic peritoneum (19%), appendiceal serosa (19%), and diaphragm (13%) [2–13]. In the present case, the lesion was found in the pelvic peritoneum. Although no history of ovarian masses or malignancy was reported, a similar pigmented lesion was incidentally discovered on the right ovary during surgery, in association with an ovarian cyst.

In all published cases of peritoneal melanosis, an associated cystic or solid tumor has been identified. Most cases were linked to benign lesions, including ovarian dermoid cysts (*n* = 4), mucinous cystadenomas of the ovary (*n* = 2), enteric duplication cysts (*n* = 2), serous cystadenoma of the ovary (*n* = 1), gastric triplication (*n* = 1), and melanotic peritoneal cyst (*n* = 1), accounting for a total of 11 benign cases [2, 4, 7–10, 13–17]. Only five cases were associated with malignancy: mucinous adenocarcinoma of the ovary (*n* = 1), serous carcinoma of the ovary (*n* = 1), adenocarcinoma of the sigmoid colon (*n* = 1), adenocarcinoma of the rectum (*n* = 1), and metastatic melanoma to the spleen *(n* = 1) [1, 3, 5, 11, 12].

Notably, one reported case involved the coexistence of a mucinous cystadenoma of the ovary and an adenocarcinoma of the sigmoid colon [6].

The macroscopic and microscopic findings in our patient were consistent with previously reported cases [10]. Histological examination of the peritoneal lesion revealed fibro-adipose stroma containing dense polymorphic inflammatory infiltrate and aggregates of pigment-laden macrophages with fine yellowish-brown granules. Some deposits showed partial calcification and a festooned arrangement beneath the mesothelial lining.

The etiopathogenesis of peritoneal melanosis remain unclear [11]. Several mechanisms have been proposed. One leading hypothesis suggests that it results from rupture or leakage of congenital cystic lesions, particularly ovarian mature teratomas containing gastric-type mucosa [2]. Peptic ulceration within these cysts may cause hemorrhage and spillage of iron-rich contents into the peritoneal cavity, leading to subsequent phagocytosis by macrophages. Alternative hypotheses include developmental anomalies, such as ectopic neural crest cell migration or differentiation of multipotent coelomic epithelial remnants into pigment-producing cells [8, 15]. In malignancy-associated cases such as colorectal adenocarcinoma or ovarian mucinous carcinoma, pigment deposition may result from tumor regression or direct release of melanin-like substances [12]. In the present case, the pigment is likely related to micro-leakage from the small ovarian lesion incidentally discovered during surgery.

Macroscopically, the main differential diagnosis is metastatic melanoma. However, microscopic examination demonstrated the absence of atypical epithelioid or spindle-shaped melanocytic cells [13]. Other mimics, including peritoneal hemosiderosis, endometriosis, deciduosis, or iatrogenic tattooing were readily excluded on histology in this case.

Peritoneal melanosis is benign and does not impact the surgical management of the primary condition [3]. However, its incidental discovery requires a systematic search for occult associated lesions [8]. Gynecologic imaging and, if indicated, ovarian exploration is recommended, given the strong historical link with ovarian lesions [3].

This case expands the limited literature on peritoneal melanosis and highlights the importance of awareness of this rare benign condition among surgeons and pathologists. Because of its macroscopic appearance, it may be confused with metastatic melanoma, leading to diagnostic confusion, unnecessary investigations, and significant patient anxiety. It is important we continue reporting additional cases, supported by advanced molecular analysis to clarify the pathogenesis and to better define appropriate follow-up strategies.
